# A European Perspective on the German System for Thrombectomy in Stroke Patients

**DOI:** 10.1007/s00062-021-00999-2

**Published:** 2021-03-09

**Authors:** Aymeric Rouchaud, Mohammed Aggour, Elisa Ciceri, Mario Martínez-Galdámez, Anne-Christine Januel, Vladimir Kalousek, Zsolt Kulcsár, Kirill Orlov, Jens Fiehler

**Affiliations:** 1 Neuroradiology Department, Dupuytren, University Hospital of Limoges, Limoges Cedex, France; 2 Department of Interventional Neuroradiology, The Royal London Hospital, London, UK; 3 Department of Neuroradiology, Azienda Ospedaliera Universitaria Integrata Borgo Trento, Verona, Italy; 4 Department of Interventional Neuroradiology, IRCCS Foundation Neurological Institute ‘C. Besta’, Milano, Italy; 5 Interventional Neuroradiology/Endovascular Neurosurgery, Hospital Clínico Universitario de Valladolid, Valladolid, Spain; 6 Department of Interventional Neuroradiology, CHU Toulouse, Toulouse, Midi-Pyrénées France; 7 Department of Radiology, Clinical Hospital Centre ‘Sestre Milosrdnice’, Zagreb, Croatia; 8 Department of Neuroradiology, Zurich University Hospital, Zurich, Switzerland; 9 Research Center of Endovascular Neurosurgery, Federal Center of Brain and Neuro Technologies, Moscow, Russian Federation; 10 Department of Neuroradiology, University Medical Center Hamburg-Eppendorf, Hamburg, Germany

Unlike the Unites States of America and China, Europe consists of multiple individual countries. Since ancient times, different national traditions and approaches competed with each other. This unique feature made Europe a test laboratory and it applies to neurointerventions as well. The European Society of Minimally Invasive Neurological Therapy (ESMINT) represents more than 40 European countries, some of them with very few neurointerventionalists some with hundreds. Many of our colleagues work in metropolitan areas, some in sparsely populated regions. Some have established national regulations for a long time for neurointerventions. Some are just starting the practice.

The paper from Rohde et al. reflects such a unique European approach [9]. The practice patterns in Germany are in line with the European guidelines for thrombectomy, which have been endorsed by the Germany Society of Neuroradiology (DGNR) directly after publication [4, 13]. In an unparalleled effort the authors managed to gather data from 13,840 thrombectomy treatments in 158 participating centers—from a single year—resulting in a thrombectomy rate of at least 8.0% for all ischemic strokes. Although similar registries have been established in other countries as well [6, 8], the authors and organizers of this database deserve praise for this impressive achievement. In this editorial, we would like to put the data into a European perspective and address some of its most relevant implications.

Firstly, how did the organizers manage to get 158 participating centers to contribute their data? Do Germans genuinely love typing in data into case record forms? The true answer is that the participation in this database was made mandatory for stroke unit certification by the German Stroke Organization (DSG) and represents a precondition for individual certificates for physicians for neurovascular recanalization (module E) and neurovascular embolization (module F) [1]. Starting in a few pilot centers, this system gained more and more momentum and increased the number of participating centers continuously over the years.

Secondly, why are there 158 centers offering thrombectomy procedures in Germany? Actually, there are even more centers who just do not participate. The resulting average catchment population of approximately 500,000 patients per center is similar to the USA with 577 centers for 309 million inhabitants [11]. In contrast, this catchment population is considerably smaller than what is observed in other European countries such as the Netherlands with a catchment population for 1 center of 1.1 million inhabitants [6], in France with 1.5 million [5] or in Italy with 2.4 million [8, 10]. One possible explanation is the decentralized federal system in Germany that fosters various organization approaches organized bottom-up. This bottom-up experimentation led to some successful organizational models, such as the drip-and-drive approach [3, 12]. On the other hand, the resulting relatively small case load in some centers might lead to inferior treatment results [2, 7]. Rohde et al. avoided such per center analyses, probably because of the expected inconclusive results from partially incomplete clinical data.

Thirdly, although the authors give valuable insights and offer benchmarks for procedural and logistical data, the scientific value of this large database has its limitations when considered as a clinical study. Accepting incomplete entries of some variables has been a conscious decision by the authors to keep the data entry as simple and fast as possible and to maintain the motivation to participate. There is incomplete information on many important data such as ASPECTS (available in 34%) and the discharge NIHSS score (available in 62%) and the missing information on the modified Rankin scale (mRS). Nevertheless, we can get many important insights such as the current rates of extracranial or intracranial stenting (8.9% and 3.4%, respectively), the rates of vessel occlusion in the anterior and posterior circulation (87.4% and 10.7%, respectively), and the observed time from symptom onset to the intervention in secondary referrals (drip and ship vs. mothership with 75 min delay). Moreover, even 34% of the overall cohort represents a substantial 5129 patients—within a single year. In contrast, the complementary German Stroke Registry with many overlapping centers reports clinical outcome data of approximately 90% of the patients (mRS at 90 days) [14].

In summary, a high number of German thrombectomy centers with various treatment volumes are participating in this highly successful and very large database. Participation is based on incentives, such as personal and institutional certification. Procedural and logistical data from such databases can be highly beneficial, particularly for organizational purposes and quality management. The authors deserve great respect for creating and reporting this successful model for collaboration among numerous hospitals.
